# Altered microRNA profile during fracture healing in rats with diabetes

**DOI:** 10.1186/s13018-020-01658-x

**Published:** 2020-04-07

**Authors:** Shunsuke Takahara, Sang Yang Lee, Takashi Iwakura, Keisuke Oe, Tomoaki Fukui, Etsuko Okumachi, Michio Arakura, Yoshitada Sakai, Tomoyuki Matsumoto, Takehiko Matsushita, Ryosuke Kuroda, Takahiro Niikura

**Affiliations:** 1 Department of Orthopaedic Surgery, Kobe University Graduate School of Medicine, 7-5-1 Kusunoki-cho, Chuo-ku, Kobe, 650-0017 Japan; 2 Department of Orthopaedic Surgery, Hyogo Prefectural Kakogawa Medical Center, Kakogawa, 675-8555 Japan; 3 Department of Orthopaedic Surgery, Showa University School of Medicine, Tokyo, 142-8666 Japan; 4 Division of Rehabilitation Medicine, Kobe University Graduate School of Medicine, Kobe, 650-0017 Japan

**Keywords:** Diabetes mellitus, MicroRNA, Fracture healing, Delayed union, Nonunion

## Abstract

**Background:**

MicroRNAs (miRNAs) are a class of small non-coding RNA molecules that regulate gene expression. There is increasing evidence that some miRNAs are involved in the pathology of diabetes mellitus (DM) and its complications. We hypothesized that the functions of certain miRNAs and the changes in their patterns of expression may contribute to the pathogenesis of impaired fractures due to DM.

**Methods:**

In this study, 108 male Sprague–Dawley rats were divided into DM and control groups. DM rats were created by a single intravenous injection of streptozotocin. Closed transverse femoral shaft fractures were created in both groups. On post-fracture days 5, 7, 11, 14, 21, and 28, miRNA was extracted from the newly generated tissue at the fracture site. Microarray analysis was conducted with miRNA samples from each group on post-fracture days 5 and 11. The microarray findings were validated by real-time polymerase chain reaction (PCR) analysis at each time point.

**Results:**

Microarray analysis revealed that, on days 5 and 11, 368 and 207 miRNAs, respectively, were upregulated in the DM group, compared with the control group. The top four miRNAs on day 5 were miR-339-3p, miR451-5p, miR-532-5p, and miR-551b-3p. The top four miRNAs on day 11 were miR-221-3p, miR376a-3p, miR-379-3p, and miR-379-5p. Among these miRNAs, miR-221-3p, miR-339-3p, miR-376a-3p, miR-379-5p, and miR-451-5p were validated by real-time PCR analysis. Furthermore, PCR analysis revealed that these five miRNAs were differentially expressed with dynamic expression patterns during fracture healing in the DM group, compared with the control group.

**Conclusions:**

Our findings will aid in understanding the pathology of impaired fracture healing in DM and may support the development of molecular therapies using miRNAs for the treatment of impaired fracture healing in patients with DM.

## Background

Diabetes mellitus (DM) is a major public health concern that is approaching epidemic proportions globally. There are 425 million people worldwide who have DM, and this number is expected to increase to 629 million by 2045 [1]. DM is associated with an extensive list of complications involving nearly every tissue in the body, including the heart, blood vessels, eyes, kidneys, and nerves. It has been reported that DM adversely affects bone health and is associated with reduced bone mineral density or reduced bone strength, resulting in a higher risk of fracture [2]. Clinical studies have shown higher incidences of delayed union and nonunion, as well as a doubling of the time to healing of fractures in patients with DM, compared with healthy patients [3, 4]. In patients with DM, fracture healing time is prolonged by up to 87% [3]. In addition, Hernandez et al. reported higher odds of delayed union in patients with DM [4]. Despite reports of associations between DM and impaired fracture healing, including delayed union and nonunion, only a few investigations have examined the molecular mechanisms by which DM affects the process of fracture healing [5–7].

microRNAs (miRNAs) are short, single-stranded, non-coding RNA molecules that regulate gene expression [8]. miRNAs play pivotal roles in biological processes, including cellular differentiation, cell growth, and organ development [9]. In the field of skeletal biology, several studies showed that miRNAs contribute to the regulation of osteoblast, chondrocyte, and osteoclast differentiation and function, suggesting that miRNAs have important roles in bone formation, resorption, remodeling, and repair [10–12]. In addition, the involvement of miRNAs in fracture healing and nonunion has been demonstrated [10, 13–18].

Recently, some miRNAs were reported to be involved in the pathology of DM and its complications [19–21]. Chronic inflammation is an important determinant of insulin resistance and contributes to microvascular complications of type 2 DM, such as nephropathy, neuropathy, and retinopathy. miR-146a has been reported to regulate genes involved in the pathogenesis of type 2 DM and its related complications by attenuating inflammatory cytokine production in macrophages [20]. Grieco et al. reported hyperexpression of miR-148a and miR-21-5p in serum/plasma from patients with type I DM. miR-148a and miR-21-5p target pivotal genes involved in bone development, modeling, and remodeling (e.g., wingless-type MMTV integration site family, member 1 (WNT1), and transforming growth factor-β 2 (TGF-β2)), thus suggesting that miR-148a and miR-21-5p serve as contributing factors to bone fragility or as potential biomarkers of bone metabolic alterations in patients with type 1 DM [21]. However, only a few studies have shown direct roles for miRNAs in impaired fracture healing in patients with DM or in animal models of DM [22].

The elucidation of miRNA expression changes during fracture healing may reveal valuable information regarding the pathophysiology of the healing process in patients with DM and suggests potential therapeutic interventions to accelerate fracture healing. This study was conducted to examine miRNA expression profiles during fracture healing of the femur of rats with DM by using microarray analysis and to elucidate the dynamic expression patterns of expressed miRNAs during fracture healing by using real-time polymerase chain reaction (PCR) analysis.

## Methods

### Animals

A total of one hundred and eight 10-week-old male Sprague–Dawley rats (SLC, Hamamatsu, Japan) were used in this study; 54 were assigned to the DM group and 54 to the control group. We created type I DM rats, as an impaired fracture healing model, by a single intraperitoneal injection of 40 mg/kg streptozotocin (STZ) (Sigma-Aldrich, St. Louis, MO, USA) [6, 7]. Rats with blood glucose levels over 300 mg/dl at 1 week after injection of STZ were used for this study as DM rats. Control rats with normal blood glucose levels were injected with sodium chloride as a sham treatment. All animal procedures were approved by the Animal Care and Use Committee of Kobe University Graduate School of Medicine (approval number: P131204).

### Surgical procedure

Closed transverse femoral shaft fractures in both groups were produced as previously described, via insertion of a 1.25-mm-diameter Kirschner wire into the right femoral intramedullary canal and induction of a fracture using a three-point bending apparatus with a drop weight [7, 23]. Preoperatively, we injected medetomidine (0.15 mg/kg), midazolam (2 mg/kg), and butorphanol (2.5 mg/kg) intraperitoneally for anesthesia and sedation. Unprotected weight bearing was allowed postoperatively. At each time points of assessment, all animals were anesthetized by isoflurane and euthanized by intraperitoneal injection of fatal dose pentobarbital sodium unconsciously.

### Radiographic assessment of fracture healing

On post-fracture days 7, 14, 21, and 28, eight animals in each group were killed, and radiographs of the fracture site were acquired. Each callus on the four cortices (two from anteroposterior and two from lateral views) was evaluated by two orthopedic surgeons blinded to animal grouping. A bony union was defined when three of four cortices were bridged and/or fracture lines disappeared completely [24].

### Strategy for miRNA expression analysis

**Fig. 1:**
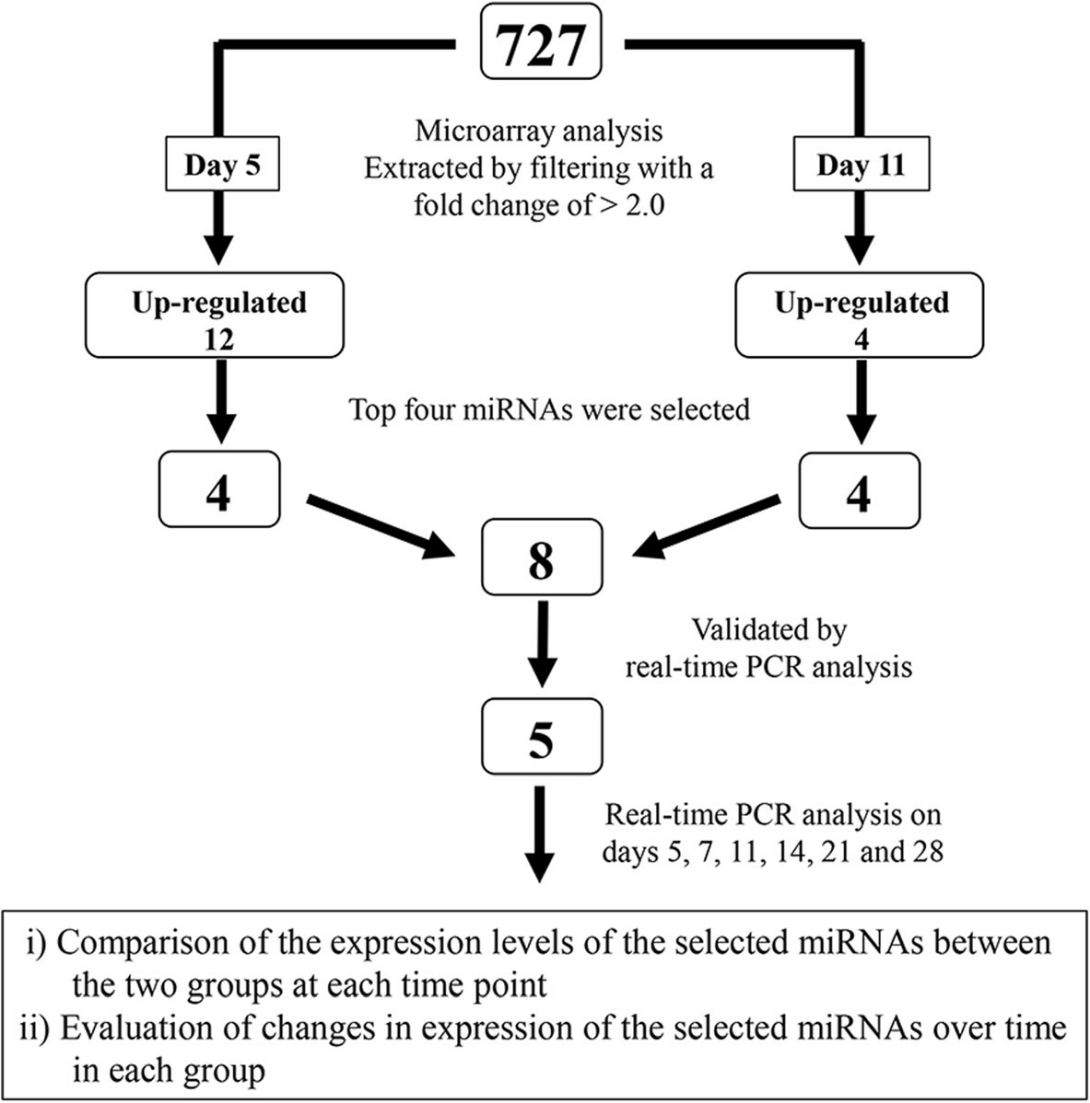
Flow chart of the experimental design. The boxes indicate microRNA (miRNA) numbers; PCR, polymerase chain reaction

Figure 1 shows the experimental design for miRNA expression analysis. As a first step, on post-fracture days 5 and 11, extracted miRNAs from the calluses from both groups were screened by microarray analysis for 727 miRNAs to identify those expressed at high levels in the DM group, compared with the control group. miRNAs were extracted by filtering with a fold change of > 2.0 or coefficient variation (< 50%) and high Hy3 signal (> 10) [13, 14]. Subsequently, these results were validated by real-time PCR analysis. This process was performed to compare the expression levels of selected miRNAs between the two groups at each time point and to investigate their changes in expression over time in each group.

### RNA extraction and miRNA microarray analysis

On post-fracture days 5 and 11, five animals in each group were killed, and the fractured femurs were harvested. Total RNA, including miRNAs, was extracted from the newly generated calluses around the fracture site using the miRNeasy Mini Kit (Qiagen, Venlo, Netherlands). With the 3D-Gene miRNA Labeling Kit (Toray, Kamakura, Japan), extracted total RNA was labeled. Labeled RNAs were hybridized onto 3D-Gene Rat miRNA Oligo chips (Toray). The annotation and oligonucleotide sequences of the probes were conformed in the miRBase miRNA database (http://www.mirbase.org/). After stringent washes, fluorescent signals were scanned with the 3D-Gene Scanner (Toray) and analyzed using the 3D-Gene Extraction software (Toray).

### Real-time PCR analysis

Based on the array results, the top four miRNAs on days 5 and 11 were selected for further real-time PCR analysis. The newly generated calluses collected on post-fracture days 5, 7, 11, 14, 21, and 28 were harvested (*n* = 6 per group at each time point). The calluses were homogenized, and total RNA was extracted. RNA was reverse-transcribed to single-stranded cDNA using the miRCURY LNA Universal RT microRNA PCR Kit (Exiqon, Vedbaek, Denmark). Real-time PCR analysis was performed in duplicate with a StepOne Sequence Detector (Applied Biosystems, Branchburg, NJ, USA), using SYBR Green Master Mix and microRNA LNA PCR primer sets (both from Exiqon). As an internal control gene in miRNA PCR assays, U6 was used [13, 14]. The conditions of the PCR amplification were 95 °C for 30 sec, followed by 40 cycles of 95 °C for 30 sec and 75 °C for 15 sec. Changes in miRNA expression levels were calculated using the comparative ΔΔCT method [13, 14] and is presented as the fold change relative to the level of the corresponding miRNA in the control group on post-fracture day 5.

### Statistical analysis

All quantitative data are expressed as means ± standard errors and were analyzed by the SPSS 18.0 software (SPSS Inc., Chicago, IL, USA). Fisher’s exact test was used for radiographic assessment of the union rates between the groups at each time point. The values of the DM and control groups were compared at each time point using the Mann–Whitney *U* test. The Kruskal–Wallis test and Mann–Whitney *U* test with Bonferroni correction were used to compare values among time points in each group. A *p* value less than 0.05 was defined as statistically significant.

## Results

### Radiographic assessment of fracture healing

**Fig. 2:**
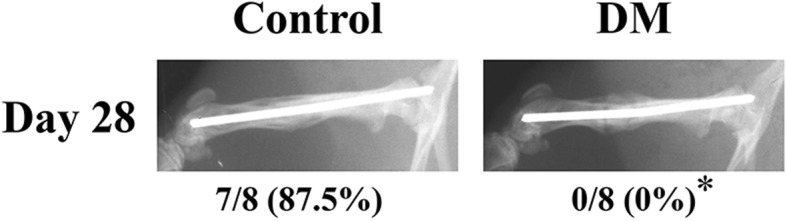
Radiographs of femurs during fracture healing in the control and DM groups at day 28. Representative radiographs are shown, and the proportion of rats with fracture union is indicated at the bottom of images. *p < 0.05 compared with the control group

Table 1 presents the union rate by radiographic assessment. On day 21, enlargement of the callus was observed, and three (37.5%) animals had achieved fracture union in the control group. On day 28, in the control group, seven (87.5%) of the animals had achieved union. On the other hands, there were no animals achieving union in the DM group on days 21 and 28. Moreover, remodeling processes were observed in the control group (Fig. 2).

**Table 1:** Union rate by radiographic assessment

	Control	DM
Day 7	0/8 (0%)	0/8 (0%)
Day 14	0/8 (0%)	0/8 (0%)
Day 21	3/8 (37.5%)	0/8 (0%)
Day 28	7/8 (87.5%)	0/8 (0%)*

*p < 0.05 compared with the control group

### miRNA microarray analysis

As presented in Fig. 1, using a miRNA-based array screening, we tested the expression levels of 727 rat miRNAs. On days 5 and 11, 368 and 207 miRNAs were upregulated in the DM group compared with the control group. Among the upregulated miRNAs, those that were highly upregulated (greater than twofold change) were extracted by filtering. Using this threshold, we identified 12 miRNAs that were highly upregulated on day 5 and four miRNAs that were highly upregulated on day 11, respectively. The top four miRNAs on day 5 were miR-339-3p, miR451-5p, miR-532-5p, and miR-551b-3p (Table 2). The top four miRNAs on day 11 were miR-221-3p, miR376a-3p, miR-379-3p, and miR-379-5p (Table 3). These data were deposited in the NCBI Gene Expression Omnibus with the accession no. GSE76365.

**Table 2:** Highly up-regulated miRNAs in DM group on post-fracture day 5 and their validated putative genes

miRNA	Fold changeDM/control	Target genes
miR-451-5p	3.44	14-3-3ζ [25], Rab5a [25]
miR-532-5p	3.23	CXCL2
miR-551b-3p	3.04	TMPRSS4
miR-339-3p	2.50	ANXA3 [26]

*CXC2L* chemokine ligand 2, *TMPRSS4* transmembrane protease serine 4,

*ANXA3* Annexin A3

**Table 3:** Highly upregulated miRNAs in DM group on post-fracture day 11 and their putative target genes

miRNA	Fold change DM/control	Target genes
miR-379-3p	2.68	Unknown
miR-376a-3p	2.09	BMPR2 [27], TGFBR1 [27]
miR-221-3p	2.03	SDF-1 [25], c-kit [28]
miR-379-5p	2.02	ATF3 [29]

*BMPR2* bone morphogenetic protein receptor 2, *TGFBR1* transforming growth factor beta receptor 1, *SDF-1* stromal cell derived factor-1

### Selection of five miRNAs for real-time PCR analysis

**Fig. 3:**
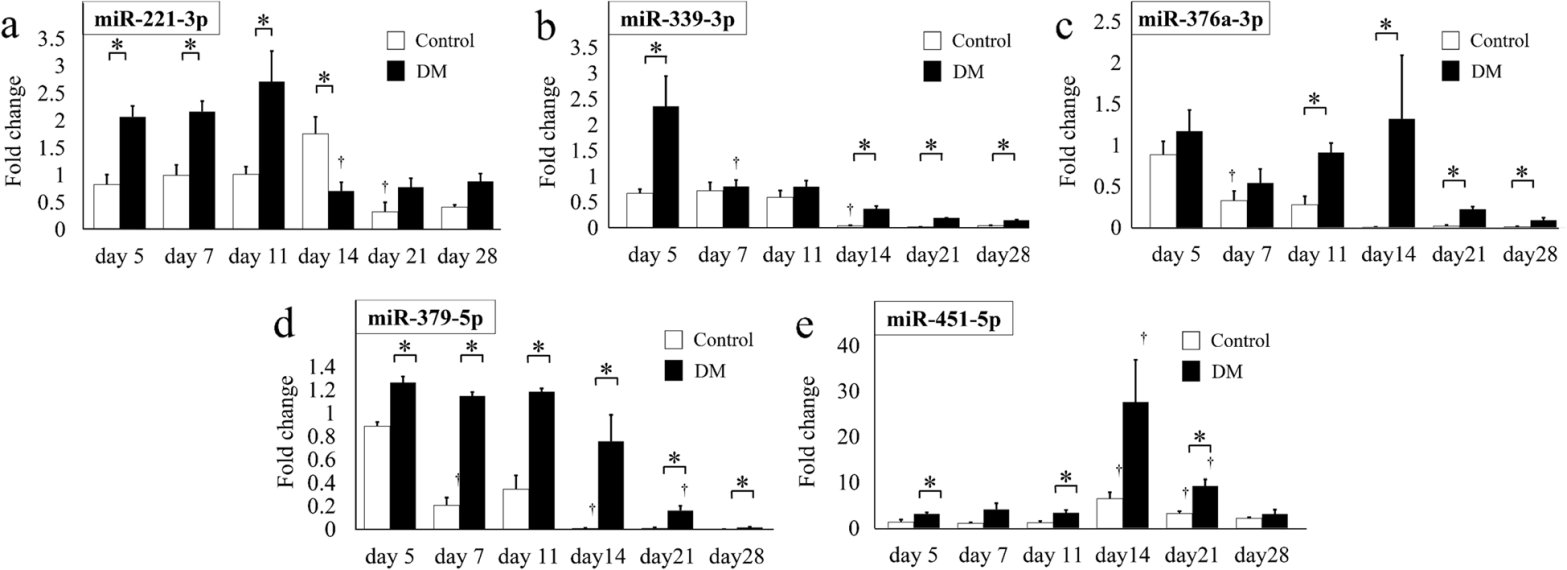
Expression of selected miRNAs, miR-221-3p (**a**), miR-339-3p (**b**), miR-376a-3p (**c**), miR-379-5p (**d**), and miR-451-5p (**e**), in the control group (blank bars) and DM group (solid bars) on post-fracture days 5, 7, 11, 14, 21, and 28 as analyzed by real-time PCR. All graphs show the fold change in expression when the expression in the control group on day 5 was normalized as 1. Values are the mean ± standard error (*n* = 6 in each group at each time point). *p < 0.05 for indicated groups at the same time point. †, *p* < 0.05 versus values on the former time point in the same group

Five of the eight miRNAs, namely, miR-221-3p, miR-339-3p, miR-376a-3p, miR-379-5p, and miR-451-5p, could be validated by real-time PCR analysis. Figure 3 presents the expression levels of these miRNAs in both groups over time.

*miR-221-3p.* miR-221-3p is reportedly associated with the stromal cell-derived factor 1 (SDF-1)/CXC chemokine receptor 4 (CXCR4) pathway and angiogenesis (Table 3) [28, 30]. The expression levels of miR-221-3p on days 5, 7, and 11 were significantly higher in the DM group than in the control group (Fig. 3a). In the control group, miR-221-3p expression peaked on day 14 and significantly decreased on day 21. In contrast, miR-221-3p expression in the DM group peaked on day 11 and significantly decreased on day 14. The expression level peaked earlier in the DM group than in the control group.

*miR-339-3p.* miR-339-3p is reportedly associated with inflammation (Table 2) [26]. The miR-339-3p expression level on day 5 was significantly higher in the DM group than in the control group, whereas the expression levels on days 14, 21, and 28 were significantly lower in the DM group than in the control group (Fig. 3b). In the control group, the miR-339-3p expression level remained unchanged from day 5 to day 11 and significantly decreased on day 14. The expression level in the DM group peaked on day 5 and then declined over time.

*miR-376a-3p and miR-379-5p.* miR-376a-3p and miR-379-5p are associated with chondrogenesis (Table 3) [27, 29]. The expression level of miR-376a-3p in the control group peaked on day 5 and then declined over time. The expression levels on days 11, 14, 21, and 28 were significantly higher in the DM group than in the control group (Fig. 3c). The miR-379-5p expression level at each time point was significantly higher in the DM group than in the control group. In the DM group, the expression level remained unchanged from day 5 to day 11 and then decreased (Fig. 3d).

*miR-451-5p.* miR-451-5p is associated with angiogenesis and inflammation (Table 2) [25, 31]. miR-451-5p expression levels on days 5, 11, and 21 were significantly higher in the DM group than in the control group (Fig. 3e). In the control and DM groups, the expression level peaked on day 14 and significantly decreased on day 21.

## Discussion

Recently, miRNAs have emerged as key regulators in the complex process of fracture healing [13–15]. In addition, some miRNAs are reportedly involved in the pathology of DM and its complications [19–21]. To better understand the underlying pathogenesis of impaired fracture healing in DM patients, we focused on miRNA expression profiles in fracture healing of the femur in diabetic rats. In the current study, we identified five miRNAs, miR-221-3p, miR-339-3p, miR-376a-3p, miR-379-5p, and miR-451-5p, which were expressed differentially with changing patterns during fracture healing in diabetic rats when compared with control rats. These findings provide new insights into the cellular processes involved in impaired fracture healing in patients with DM.

Bone fracture healing involves remarkably complex processes that require the coordination of a sequence of many biological events [32]. The rationale for the selection of two time points, post-fracture days 5 and 11, for microarray analysis was that key cellular events of the fracture healing process in healthy rats take place on days 5 and 11. The process of fracture healing can be divided into three overlapping phases: inflammation, repair, and remodeling [32]. Post-fracture day 5 corresponds to the transitional period from the inflammatory phase to the repair phase [33]. Post-fracture day 11 corresponds to the repair phase, which involves the processes of intramembranous and endochondral ossifications, including osteogenesis, chondrogenesis, and vascular invasion [33]. Imbalance in inflammatory responses, reduced proliferation and differentiation of osteoblasts and chondrocytes, and reduced function and alteration in vascularization have been implicated as plausible pathogenic mechanisms underlying impaired fracture healing in patients with DM [2]. Previous studies showed that long-bone fractures of STZ-induced diabetic animals result in smaller calluses with reduced bone and cartilage formation, reduced proliferation and differentiation of osteoblasts and chondrocytes, and reduced mechanical strength [6, 7, 34]. In agreement with these previous studies, radiographic assessment in the present study showed that the fracture healing process in the DM group was delayed at days 21 and 28, compared with healing in the control group. Histological evaluation also revealed a delayed fracture healing process in the DM group, which was characterized by a smaller cartilage callus and a delay in endochondral ossification (Supplemental materials).

Inflammation is a critical factor during fracture healing, with inflammatory cells and molecular factors appearing locally at the fracture site in a distinct spatial and temporal manner [33]. Highly regulated inflammatory signaling during fracture healing is essential for priming bone regeneration. Disturbances in the finely tuned inflammatory responses at the fracture site lead to impaired vascularization, reduced bone formation, disturbed osteoclastic function, and subsequent delayed fracture healing or nonunion [35]. miR-339-3p was reported to inactivate the Akt/mammalian target of rapamycin (mTOR) signaling pathway and to alleviate inflammation and edema caused by severe acute pancreatitis with acute lung injury in a mouse model by targeting annexin A3 (ANXA3) [26]. In addition, rapamycin, an inhibitor of mTOR, inhibits osteogenesis both in vitro and in vivo [36]. Inhibition of mTOR by rapamycin blocked insulin-like growth factor 1-induced osteoblast differentiation of mesenchymal stem cells and mineralization [36]. miR-451-3p reduces inflammation by suppressing phosphorylation of p38 mitogen-activated protein kinase (MAPK) via 14-3-3ζ and Rab5a [25]. p38 MAPK plays an important signaling role in orchestrating injury or stress-induced responses, as well as in bone formation [37]. Pro-inflammatory cytokines, such as tumor necrosis factor-α and interleukin (IL)-1a, activate p38 MAPK; activation and signaling of p38 MAPK also lead to the production of these pro-inflammatory cytokines and their signal transduction. The interactions between p38 MAPK and pro-inflammatory cytokines are important for controlling life and death signaling cascades in osteoblasts and chondrocytes. Taken together, miR-339-3p and miR-451-3p may play important roles during fracture healing by regulating inflammation.

Chondrogenesis is an essential component of endochondral ossification in fracture healing [32]. miR-376a-3p is one of the most important miRNAs to trigger chondrogenesis [27]. Bakhshandeh et al. [27] reported that the expression of miR-376a was downregulated during the early phases of chondrogenic differentiation in unrestricted somatic stem cells. Bone morphogenetic protein (BMP) receptor 2 and TGF-β receptor 1 are putative target genes of miR-376a [27]. Many studies have demonstrated that BMP-2 induces chondrogenic differentiation in various types of stem cell [38]. The recent study revealed that BMP-2 and TGF-β1 cooperate during chondrogenic differentiation of synovium-derived mesenchymal stem cells [39]. In addition, miR-379-5p, which is predicted to target activating transcription factor 3 (ATF3), regulates the endochondral ossification process by modulating proliferation and hypertrophic differentiation of the growth plate in male rats [29]. James et al. demonstrated the importance of ATF3 in the control of endochondral bone growth and skeletal development [40]. Some fundamental aspects of fracture healing, particularly endochondral ossification, have clear parallels with long bone development [41]. The altered expression of miR-376a-3p and miR-379-5p at the fracture site in rats with DM might dysregulate chondrogenesis and thus inhibit endochondral ossification, which could lead to impaired fracture healing.

Angiogenesis and bone formation are coupled during fracture healing [42]. A lack of oxygen (hypoxia) in fractured bone and the subsequent generation of angiogenic factors are critical for achieving successful fracture healing [43]. miR-451-5p was reported to inhibit cell growth, migration, and angiogenesis via downregulation of IL-6R [31]. miR-221-3p regulates angiogenesis by directly reducing the levels of c-Kit [28]. c-Kit and its ligand, stem cell factor (SCF), play an important role in the proliferation and differentiation of hematopoietic progenitor cells [44]. In addition, miR-221-3p inhibits the SDF-1/CXCR4 signaling pathway in human chondrocytes [30]. The SDF-1/CXCR4 axis plays a pivotal role in fracture healing by affecting the migration and differentiation of progenitor cells at fracture sites [7, 45, 46]. Kitaori et al. [45] reported that SDF-1 was induced in fractured bone and promoted endochondral bone formation by recruiting mesenchymal progenitor cells to the site of injury. Moreover, the SDF-1/CXCR4 axis acts as a key regulator of angiogenesis and contributes to the regulation of endothelial progenitor cell (EPC) recruitment in ischemic tissues. EPCs represent a small population of blood cells that can differentiate into endothelial cells and participate in postnatal angiogenesis [47]. In addition, EPCs are involved in physiological and pathological angiogenesis/vasculogenesis [48]. Recently, Arakura et al. [7] revealed that gene expression levels and localizations of SDF-1 and CXCR4 at fracture sites were altered during fracture healing in animals with experimental DM, which may contribute to the impaired fracture healing associated with inhibition of endochondral ossification and angiogenesis. Taken together, the altered expression patterns of miR-221-3p and miR-451-5p in the DM group during fracture healing suggest that disturbed angiogenesis/vasculogenesis and endochondral ossification may occur at fracture sites, potentially leading to impaired fracture healing.

The present results have clinical implications. Due to the increasing number of patients with DM, the incidence of impaired fracture healing associated with DM is expected to increase. It is desirable to establish strategies to prevent impaired fracture healing and enhance bone healing in patients with DM. Many biological and biophysical interventions have been attempted to accelerate fracture healing in these patients, including the use of exogenous growth factors, such as bone morphogenetic proteins, low-intensity pulsed ultrasound, and stem/progenitor cell transplantation [49]. Although most of these strategies have shown relatively satisfactory results, there are some notable limitations to their effectiveness and availability. The recent discovery of miRNAs, being powerful regulators in a variety of tissues and processes, suggests their potential as therapeutic target. Several therapeutic trials targeting miRNAs have been conducted [50].

Notably, the use of miravirsen (Santaris Pharma A/S, Copenhagen, Denmark), an anti-miR-122 drug, in patients with chronic hepatitis C (HCV) infection showed prolonged dose-dependent reductions in HCV RNA levels without evidence of viral resistance [51], which implies that miRNA-based therapeutics can indeed become a reality in clinical medicine. In the field of orthopedics, Murata et al. demonstrated that inhibition of miR-92a enhanced fracture healing in mice [15]. The five miRNAs identified in our study might represent a tool for the establishment of therapeutic approaches, which either prevent impaired fracture healing or enhance fracture healing. Further in vivo functional analyses will be needed to clarify the exact role and therapeutic potential of each miRNA during fracture healing.

This study had a few limitations. First, we did not demonstrate specific regulatory activities exerted by these five miRNAs; hence, our results solely indicate associations with impaired fracture healing, rather than clear causative relationships. Further in vivo analyses, including the loss or gain of miRNA function tests, are needed to define the exact role of each miRNA during fracture healing. Second, our study did not validate target genes of the five selected miRNAs. Target identification of miRNAs is computationally difficult due to the relatively low homology between miRNAs and their targets. Further, miRNAs may target any of the listed target genes in one cell type but not in others (given the diversity of cell types found within the fracture callus), and as such many may not be related specifically to bone or cartilage cells [17]. Further investigation is needed to clarify the contributions of each miRNA identified in this study to the downregulation of specific genes in the context of fracture healing process.

## Conclusions

Altered expression levels of five miRNAs—miR-221-3p, miR-339-3p, miR-376a-3p, miR-379-5p, and miR-451-5p—may contribute to impaired fracture healing in patients with DM. These findings provide valuable information to understand the pathology of impaired fracture healing in patients with DM, and may lead to the development of therapeutic strategies using miRNA for the treatment of fractures in patients with DM.

## Supplementary information

**Additional file 1:.** Figure S1

**Additional file 2:.** Figure S2

## Data Availability

The datasets used and analyzed during the current study are available from the corresponding author on reasonable request.

## Abbreviations

DM: Diabetes mellitus
miRNA: MicroRNA
mRNA: Messenger RNA
WNT1: Wingless-type MMTV integration site family, member 1
TGF-β2: Transforming growth factor-β 2
STZ: Streptozotocin
PCR: Polymerase chain reaction
GEO: Gene Expression Omnibus
SDF-1: Stromal cell-derived factor 1
CXCR4: CXC chemokine receptor 4
mTOR: Mammalian target of rapamycin
ANXA3: Annexin A3
MAPK: Mitogen-activated protein kinase
IL: Interleukin
BMP: Bone morphogenetic protein
ATF3: Activating transcription factor 3
SCF: Stem cell factor
EPC: Endothelial progenitor cell
