# Supplementary figures and images for: Altered microRNA profile during fracture healing in rats with diabetes
Source: J Orthop Surg Res. 2020 Apr 7;15:135. doi: 10.1186/s13018-020-01658-x (PMC7140490; doi:10.1186/s13018-020-01658-x)

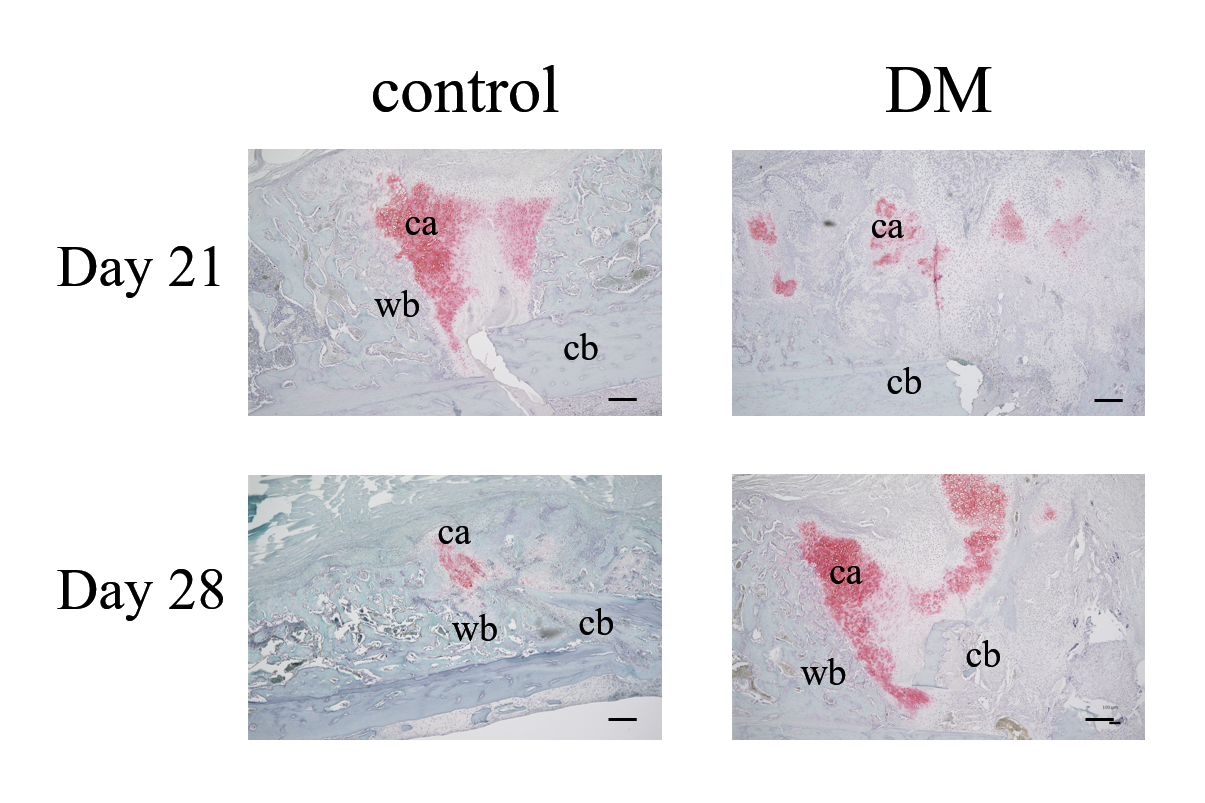

Supplement: Supplementary file 1 — Additional file 1:. Figure S1 [file 13018_2020_1658_MOESM1_ESM.tif]

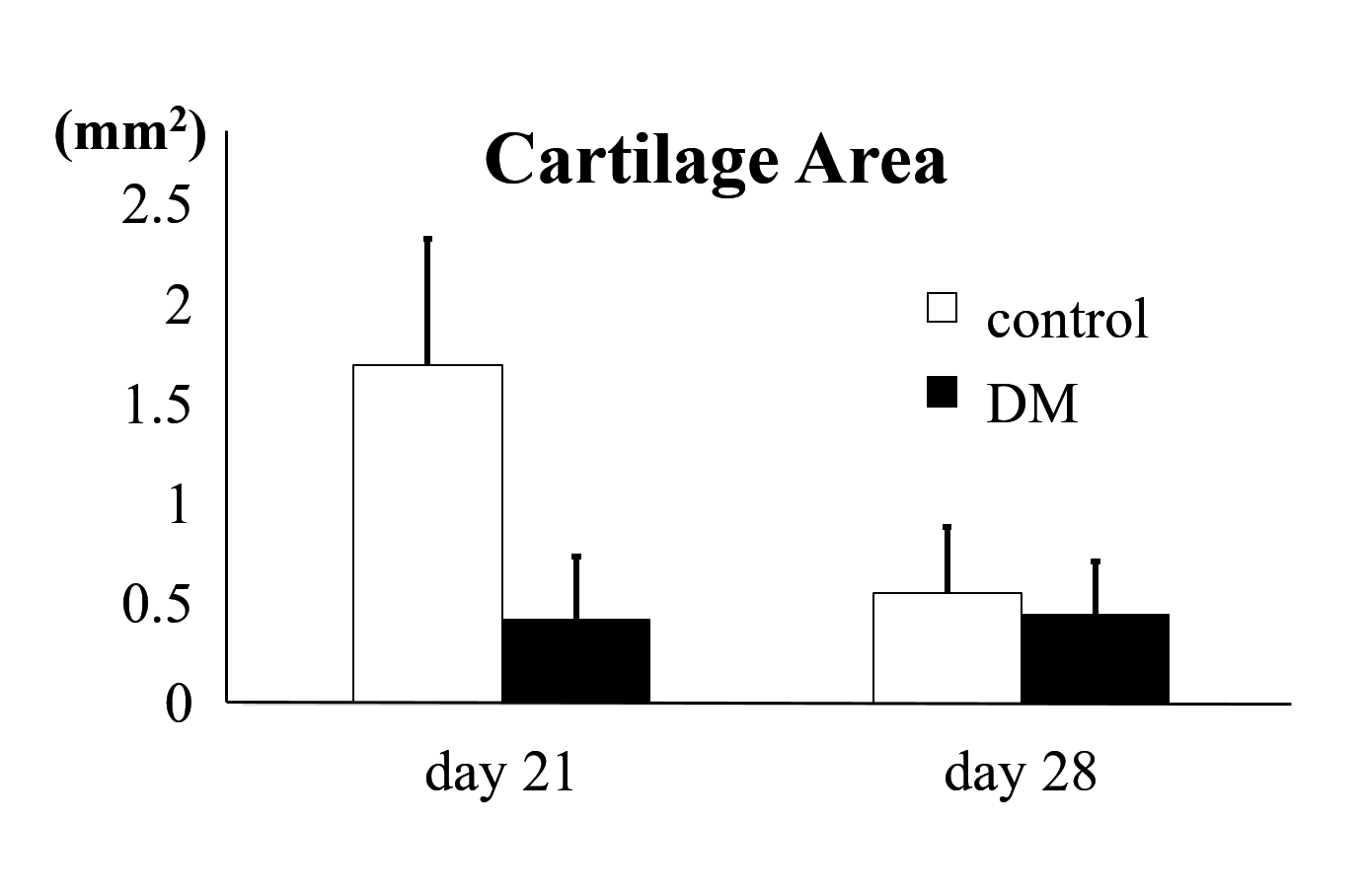

Supplement: Supplementary file 2 — Additional file 2:. Figure S2 [file 13018_2020_1658_MOESM2_ESM.tif]
